# Effects of Resistance Exercise Order on the Number of Repetitions Performed to Failure and Perceived Exertion in Untrained Young Males

**DOI:** 10.2478/hukin-2013-0080

**Published:** 2013-12-31

**Authors:** Nuno Romano, José Vilaça-Alves, Helder M. Fernandes, Francisco Saavedra, Gabriel Paz, Humberto Miranda, Roberto Simão, Jefferson Novaes, Victor Reis

**Affiliations:** 1 University of Trás-os-Montes and Alto Douro (UTAD), Vila Real, Portugal.; 2 Research Center for Sport, Health and Human Development (CIDESD), Vila Real, Portugal.; 3 School of Physical Education and Sports, Rio de Janeiro Federal University, Rio de Janeiro, Brazil.

**Keywords:** Resistance training, Exercise order, Strength performance, Adolescents

## Abstract

Exercise order is an essential variable of resistance training (RT) programs which is usually related to repetition performance. The purpose of this study was to investigate the acute effect of different resistance exercise order on the number of repetitions performed to failure and related ratings of perceived exertion (RPE). Thirteen male adolescents (age: 14.46 ± 1.39 years, body height: 165.31 ± 12.75 cm, body mass: 58.73 ± 12.27 kg, estimated body fat: 21.32 ± 2.84%), without previous experience in RT, performed four resistance exercises: incline leg press (ILP), dumbbell lunge (DL), bench press (BP) and lying barbell triceps extension (TE) in two sequences - Sequence A (SEQA): ILP, DL, BP and TE; sequence B (SEQB): ILP, BP, DL and TE. The exercise sequences were performed in a randomized crossover design with a rest interval of 72h between sessions. Within-subjects analysis showed significant differences in the number of repetitions performed to failure in both sequences, but not in the RPE. Post-hoc tests revealed significant decrements in the number of repetitions from the first to the remaining exercises in both sequences. However, pairwise comparisons did not indicate significant differences between the same exercises performed in different sequences. In conclusion, the results of the current study in adolescents suggest that the main exercises should be performed at the beginning of the RT session.

## Introduction

Resistance training (RT) has been consistently used as an efficient training method for the development of muscular strength, power, and hypertrophy (ACSM, 2009a; Folland and Williams, 2007). A primary concern of the prescription of RT should take into account the individual’s goals to be achieved (ACSM, 2011). For this reason, the interaction of loading variables should be carefully considered during the prescription of RT programs such as the type of exercise, load, number of repetitions, number of sets, type of muscular contraction, speed, rest interval between sets and exercises, and also exercise order (Miranda et al., 2010; Simão et al., 2012).

Several studies have been investigated one or more of the aforementioned variables in children and youth (Faigenbaum et al., 1999, 2008, 2009). However, to date, no study has been identified describing the effects of different exercise orders on the number of repetitions in children and/or youth. On the other hand, different authors have studied the effects of these variables among adults (Miranda et al., 2010; Sfrozo and Touey, 1996; Simão et al., 2005, 2012). For example, Sforzo and Touey (1996) concluded that multi-joint exercises should be executed before single-joint exercises in order to maximize the muscular performance as given by the total force production. In addition, Simão et al. (2005) verified the influence of different exercise orders on the number of repetitions and on the ratings of perceived exertion (RPE), and found that in both sequences the exercises performed in the end of the session resulted in significantly fewer repetitions. Nonetheless, significant differences were not found in the RPE regardless of the sequences. Noteworthily, research on the exercise sequence issue has demonstrated and recognized its importance in maximizing the results and achieving the intended goals (Gentil et al., 2007; Miranda et al., 2010; Sfrozo and Touey, 1996; Simão et al., 2010). However, no consensus has been reached yet on the optimal exercise order and the influence of this variable on strength performance and RPE during RT sessions (Simão et al., 2005, 2007; Spineti et al., 2010; Spreuwenberg et al., 2006).

Not surprisingly, in recent years increasing attention has been given to RT for children and youth by internationally renowned associations (Faigenbaum et al., 2009). Considering the importance of the development of appropriate muscular balance and strength in this period of life, it is of crucial relevance to investigate whether performing resistance exercises alternating limbs (lower and upper) may promote better repetition performance and perceived effort than performing two exercises in sequence for the same muscle group. The obtained evidence is expected to contribute to safer and more appropriate prescription programs of RT among these age groups, which then can be translated onto various practice settings where children and adolescents become or are physically active (school, sports/competition, fitness clubs, etc).

Therefore, the purpose of the current study was to investigate the effects of different exercise order on the number of repetitions performed to failure and related RPE during resistance exercises in untrained youth male subjects.

## Material and Methods

### Participants

Thirteen male adolescents (age: 14.46 ± 1.39 years, body height: 165.31 ± 12.75 cm, body mass: 58.73 ± 12.27 kg, estimated body fat: 21.32 ± 2.84%), without previous experience in RT, voluntarily participated and completed all established procedures and assessments for this study. Before participating in the study, all adolescents completed a physical activity and medical history questionnaire (ACSM, 2009b). Additionally, the participants and their parents were informed about the possible risks or discomfort involved in the experiment and provided a written informed consent form. The procedures were designed and followed according to the Helsinki Declaration and were approved by the institutional research ethics committee.

### Measures

One week before participation in the study, all participants were required to complete the following assessments.

#### Anthropometric and maturation assessment

Anthropometric measures were recorded in light clothing using a portable stadiometer (Sanny ES 2030, Physical Nutri, Araraquara, SP, Brazil) with a precision of 0.1 cm and a portable scale (Seca, Cirencester, UK) with a precision of 0.1 kg. Body fat was estimated from measurements of skinfold thicknesses as suggested by the Lohman (1987) protocol, using a skinfold caliper (Sanny AD1010, Physical Nutri, Araraquara, SP, Brazil). Sexual maturity status was self-assessed through the use of the Tanner pubertal scale (Marshall and Tanner, 1970).

#### Eight-Repetition Maximum Testing

The 8RM testing protocol followed the procedure previously described by Miranda et al. (2010). The 8RM tests were conducted in the following order: incline leg press (ILP), bench press (BP), dumbbell lunge (DL) and lying barbell triceps extension (TE). The retest was conducted 72 hours after in the reverse order: TE, DL, BP and ILP, and showed excellent reliability (intraclass correlation coefficients: ICC>0.98). All machine based exercises were performed on Life Fitness equipment (Brunswick Company, Franklin Park, Illinois, USA). During the 8RM testing, each subject performed a maximum of three 8RM attempts for each exercise, with a 5-minute rest period between trials and a 10-minute rest period between different exercises. Standard exercise techniques were given and followed for each exercise. No rest pause was allowed between the eccentric and concentric phases of repetitions. For a repetition to be successful, a complete range of motion (as normally defined) for each exercise had to be performed. All tests were preceded by a warm-up consisting of 12 repetitions with light loads. A metronome (Korg MA-30, New York, USA) was set and used at a cadence of 60 beats per minute in order to establish a rate of 30 exercise repetitions per minute.

#### Perceived Exertion

Ratings of perceived exertion were assessed using the children’s OMNI-RES scale of perceived exertion (Robertson et al., 2005) on an eleven point scale (0= extremely easy to 10= extremely hard). Standard instructions for the OMNI-RES were read to the adolescents before each testing session. Previous evidence has supported the concurrent validity of this measure in children/adolescents performing upper and lower body resistance exercises (Robertson et al., 2005).

### Procedures

Initially, prior to the commencement of the study, the subjects were submitted to two weeks of training, two sessions per week, in order to familiarize with the RT exercises performed in the current study, namely ILP, DL, BP and TE. During this familiarization period a higher emphasis was placed on learning the proper exercise techniques and brief pauses between repetitions were allowed in order to reset their starting positions when necessary (Faigenbaum et al., 2009). In the second week, participants were also measured for body mass, height and body fat percentage, and self-rated their sexual maturity status. In the same session, adolescents completed the 8 repetitions maximum (RM) loads for each exercise and then, after 72 hours, the 8RM tests were repeated to determine test-retest reliability. In these testing sessions, participants were also familiarized with the OMNI-RES scale. In the following week, the subjects participated in the experimental protocol in randomized order with an interval of 72 hours between exercise sequences.

### Exercise sessions

Two different exercise sequences were designed and composed of alternate lower and upper-body RT exercises (SEQA: ILP, DL, BP and TE) or of two exercises in sequence for the same muscle group (SEQB: ILP, BP, DL and TE). Participants performed the A or B sequence, through a randomized crossover design, at the same time of the day. Seven subjects performed SEQA first, while the remaining six subjects performed SEQB. The warm-up before each sequence consisted of 12 repetitions of each exercise, in the assigned sequence, with a 20% load of 8RM. After a three minute rest interval, adolescents performed the exercise sequence with 80% of the 8RM and with a 60-bpm cadence (rate of 30 exercise repetitions per minute). RT exercises were performed until concentric failure with a resting period of 90 seconds between exercises. Immediately after each exercise, participants reported their RPE with emphasis on local fatigue (predominantly active muscle groups). After 72 hours, all participants performed the other sequence which they were previously assigned. The procedures and instructions of the first session were maintained in the second exercise session.

### Statistical Analyses

Descriptive statistics of data were presented as mean (M) and standard deviation (SD). The normality test of Shapiro-Wilk and the homogeneity of variance and covariance were confirmed using the Levene’s test and Mauchly sphericity test. All variables presented normal distribution. Test-retest reliability was examined by using the intraclass correlation (ICC). To compare the number of repetitions performed to failure and RPE in the two sequences, one-way ANOVAs with repeated measures were used followed by post-hoc tests with Bonferroni adjustment for multiple comparisons. Paired t-tests were used to examine specific exercise differences across different sequences. The significance level was set at *p*<0.05.

## Results

Initial repeated measures ANOVA included the sexual maturity status as a covariate, but results showed no significant effects on the number of repetitions (*p*=0.083) and on the RPE (*p*=0.250). Therefore, it was excluded from further analyses.

Table 1 presents the number of exercise repetitions performed to failure in both sequences.

Within-subjects analysis showed significant differences in the number of repetitions performed to failure in SEQA (*F*_(3,36)_=9.35, *p*<0.001) and SEQB (*F*_(3,36)_=7.22, *p*=0.001). Post-hoc tests revealed significant decrements in the number of repetitions from the first to the remaining exercises in both sequences (*p*<0.05). Pairwise comparisons of the same exercises in different sequences failed to show significant differences (*p*>0.05).

The results of the RPE for each exercise according to the sequences are presented in Table 2.

Within-subjects analysis revealed no significant differences between the RPE of sequenced exercises in SEQA (*F*_(1.67, 20.04)_=0.78, *p*=0.512) and SEQB (*F*_(3,36)_=0.80, *p*=0.505). Pairwise comparisons of the same exercises in different sequences only showed a significant difference in the first exercise (ILP) of both sequences (*t*_(12)_=4.63, *p*=0.001).

## Discussion

The key finding from the current study was that different exercise order across sequences (SEQA and SEQB) did not affect the number of repetitions completed to failure. These results suggest that lower and upper body exercises, involving similar muscle groups and neural recruitment patterns, were not negatively affected in terms of repetition performance when two exercises for lower body were performed before two exercises for upper body muscles, or when the lower and upper body exercises were performed in an alternating manner. These findings may be related to the differences in the metabolic ability to recover from physical fatigue between adolescents and adults (Falk and Dotan, 2006). Indeed, it seems that the rest interval in a RT can be reduced when referring to adolescents (Faigenbaum et al., 2008) as several studies have shown that young people are able to recover from intermittent exercise of high intensity over a shorter period (Faigenbaum et al., 2008; Falk and Dotan, 2006; Zafeiridis et al., 2005).

Under such logic, Faigenbaum et al. (2008) observed the effect of a rest interval on the BP exercise performance between children, adolescents and adults. Each subject performed 3 sets of 10RM with a rest interval of 1, 2 and 3 minutes between sets. Significant differences in the performance between the different groups were observed for each rest interval. More precisely, children and adolescents performed significantly more repetitions than adults following protocols with 1 minute (27.9±3.1, 26.9±3.9 and 18.2±4.1, respectively), 2 minute (29.6±1.0, 27.8±3.5 and 21.4±4.1, respectively) and 3 minute (30.0±0.0, 28.8±2.4 and 23.9±5.3, respectively) rest intervals. These results indicate that children and adolescents have greater ability to maintain muscle performance during high intermittent intensity exercise compared with adults. Other studies have also found similar evidence (Falk and Dotan, 2006; Zafeiridis et al., 2005). Despite this idea, widely accepted among professionals, there is still little physiological information to explain this phenomenon. This faster recovery appears to be predominantly due to the fact that adolescents have a lower ability to produce energy (force), since they have a lower potential for recruitment of motor units (Falk and Dotan, 2006). Another possible reason may be associated with dimensional differences between adolescents and adults, affecting intramuscular and circulatory transient times and promoting a faster recovery (Falk and Dotan, 2006). Consequently, in the present study it is possible to conclude that in a RT session with adolescents, similar muscular performances are maintained when different exercise order sequences are performed.

However, a within sequences order effect was found in the present study when comparing the first RT exercise with the following exercises. Previous studies by Simão et al. (2005, 2007, 2012) have found the same evidence in adults and suggest that exercises performed at the end of the session tend to be negatively affected in the number of repetitions completed. Considering that, Simão et al. (2005, 2007) have suggested that the main exercises should be performed at the beginning of the RT session whether the exercise is important to develop a specific task or to increase the strength and power output of the recruited muscle groups. Simão et al. (2005) investigated the influence of different exercise orders on the number of repetitions performed in a group composed of both men and women with at least two years of recreational RT experience. The exercise sessions consisted of performing three sets of each exercise with a resistance of 10RM and two minute rest periods between sets and exercises. One session began with exercises recruiting large muscle groups and progressed towards small muscle groups exercises: BP, lat pull down (LPD), shoulder press (SP), biceps curl (BC) and TE, whereas the other session was performed with the opposite exercise sequence (TE, BC, SP, LPD, and BP). The results demonstrated performing either large or small group exercises for the upper-body at the end of an exercise sequence resulted in significantly fewer repetitions compared to when the same exercises were performed at the beginning of the exercise sequence. Moreover, Simão et al. (2007) also found a decrease in the total number of repetitions performed when both upper and lower-body exercises were performed in the same exercise sequence by 23 women with a minimum of two years of RT experience. Data were collected in two phases: determination of a 1RM and execution of three sets, with two-minute rest intervals between sets and exercises, using 80% of 1RM until fatigue in two exercise sequences of opposite order. In agreement with the previously mentioned study, Simão et al. (2007) observed that an exercise performed last in the end of a RT session was negatively affected in an acute manner whether the exercise involved large or small muscle groups. Taken together, this evidence in adults is corroborated by the results of the present study among untrained male adolescents.

With respect to the analysis of the perceived exertion ratings, the significant differences found in the ILP exercises between sequences are difficult to explain on the basis of the adopted procedures. As previously mentioned, adolescents performed the two sequences in a randomized order with the aim of excluding possible adaptation/habituation interferences and overestimation perceptions of their physical abilities in different orders. Moreover, the ILP exercise was the first to be executed in both sequences which also disregards any possible within sequences exercise order effects. As such, future research is needed to explore possible resistance exercise order effects on the rating of perceived exertion in children and adolescents of different gender and training experience, as well as other possible confounding factors. Traditionally, the RPE has been often used as an exercise intensity indicator for aerobic activities. However, recent advances in research have resulted in the development of the OMNI-Resistance Exercise Scale (OMNI-RES), which has been designed to assess RPE of RT exercises (Day et al., 2004; Tomporowski, 2001). Furthermore, recent evidence suggests that this scale (OMNIRES) can be used to determine the RPE during an exercise session of RT in adolescents (Robertson et al., 2005). With the exception of the significant difference found in the IPL exercise across sequences, the results of the present investigation demonstrated that the RPE were not altered by the exercises performed to failure. In a similar way, Simão et al. (2005, 2007) observed no significant differences in the RPE of different sequences of exercises in adults. Therefore, it is suggested that the RPE of adolescents, regardless of performing exercises for the same muscular groups consecutively or alternately, will not be affected when a rest interval of 90s between exercises is adopted. However, more research is needed to determine the sensitivity and accuracy of this instrument to changes in RT performance and fatigue in children and adolescents, as well as the number and order of RT exercises necessary to induce significant changes on the perceived exertion ratings.

## Conclusions

In conclusion, the results of the present study in untrained young males suggest that lower and upper body exercises, involving similar muscle groups and neural recruitment patterns, were not negatively affected in terms of repetition performance when two exercises for lower body were performed before two exercises for upper body muscles, or when these exercises were performed in an alternating manner. However, results demonstrated that the number of repetitions performed to failure was significantly higher for the first RT exercise, regardless of the sequence order, which suggests that adolescents should perform their main RT exercises at the beginning of the session. These findings have important implications for the prescription of ST programs in these age groups, and should be considered by PE teachers and coaches in physical education and sports practices.

## Figures and Tables

**Table 1:** Number of repetitions per exercise in both exercise sequences^*^

	
	Sequence A				Sequence B			

	ILP	DL	BP	TE	ILP	BP	DL	TE
Number of Repetitions	17.08 ± 2.29	13.62 ± 2.93	13.08 ± 2.10	14.62 ± 2.84	18.38 ± 2.60	13.85 ± 2.54	15.00 ± 2.86	16.00 ± 2.31

* Values are given as mean± SD; ILP: incline leg press; DL: dumbbell lunge; BP: bench press; TE: lying barbell triceps extension

**Table 2:** Ratings of perceived exertion per exercise in both exercise sequences^*^

	
	Sequence A				Sequence B			

	ILP	DL	BP	TE	ILP	BP	DL	TE
RPE	7.08 ± 0.76	6.62 ± 1.71	6.77 ± 1.01	6.46 ± 0.78	5.92 ± 0.86	6.31 ± 0.95	6.39 ± 1.50	6.15 ± 0.80

* Values are given as mean±SD; ILP: incline leg press; DL: dumbbell lunge; BP: bench press; TE: lying barbell triceps extension
